# Carbon-modified TiO_2 _for photocatalysis

**DOI:** 10.1186/1556-276X-7-235

**Published:** 2012-04-26

**Authors:** Malgorzata Wojtoniszak, Diana Dolat, Antoni Morawski, Ewa Mijowska

**Affiliations:** 1 Institute of Chemical and Environment Engineering, Westpomeranian University of Technology in Szczecin, Pulaskiego 10, Szczecin, 70-322, Poland

**Keywords:** Graphite, CVD, TiO_2_, Photocatalyst

## Abstract

Here we present a method to produce TiO_2 _nanocrystals coated by thin layer of graphitic carbon. The coating process was prepared via chemical vapor deposition (CVD) with acetylene used as a carbon feedstock with TiO_2 _used as a substrate. Different temperatures (400°C and 500°C) and times (10, 20, and 60 s) of reaction were explored. The prepared nanocomposites were investigated by means of transmission electron microscopy, Raman spectroscopy, thermogravimetric analysis, Fourier transform infrared spectroscopy/diffuse reflectance spectroscopy and ultraviolet-vis (UV-vis)/diffuse reflectance spectroscopy. Furthermore, photocatalytic activity of the materials was investigated under visible and UV-vis light irradiation in the process of phenol decomposition. It was found that TiO_2 _modification with carbon resulted in a significant increase of photoactivity under visible irradiation and decrease under UV-vis light irradiation. Interestingly, a shorter CVD time and higher process temperature resulted in the preparation of the samples exhibiting higher activity in the photocatalytic process under visible light irradiation.

## Background

Titanium dioxide generates a great interest in materials science due to its amazing photocatalytic performance, low cost, long-term stability, and promising application in photocatalysis areas such as wastewater [1] and air purification [2], degradation of brevetoxins in aqueous and organic media [3], or destruction of microorganisms in water [4]. Unfortunately, due to its relatively high band-gap energy (3.0 eV for rutile and 3.2 eV for anatase), TiO_2 _can be excited only by ultaviolet (UV) light. That is why the modification of TiO_2 _towards shifting the absorption threshold to the visible light region in order to allow utilization of solar energy attracted attention of many researchers. Therefore, several methods of TiO_2 _modification have been proposed such as semiconductors coupling [5], doping [6], or fluorination [7]. These methods resulted in enhanced photocatalytic activity under visible light irradiation.

Recently, graphene, a single-atom planar sheet of sp^2^-bonded carbon atoms, has attracted a great interest in the field of photocatalysis. The attention comes from its outstanding properties. For instance, graphene has a charge mobility which is among the highest of any other semiconductors [8-10] is the strongest material ever measured [11] and has a thermal conductivity more than double that of diamond [12]. Furthermore, its high surface area to volume ratio makes graphene an ideal support for TiO_2_. It was found that titanium dioxide functionalized with graphene exhibits enhanced photocatalytic performance under visible and UV light irradiation in comparison to the pristine material [13]. Graphene can improve an efficiency of photo-conversion since it may act as an electron transfer channel and inhibits a recombination of the electron-hole pairs. So far, many methods have been developed in order to synthesize graphene-modified TiO_2 _photocatalysts [14,15]. Wang et al. [14] synthesized TiO_2_-graphene nanocomposite from melamine which was used as a precursor of graphene. In the report of Zhang et al. [13], graphene was obtained from graphene oxide and further used as TiO_2 _support in the nanocomposite. In our study, we developed a method to produce TiO_2 _/graphitic carbon nanocomposite using chemical vapor deposition (CVD). Here, CVD process of acetylene used as graphene source with TiO_2 _as a template was performed. The obtained material was further used as a photocatalyst in the phenol decomposition under UV-vis and visible light irradiation.

## Methods

### Materials

Pristine TiO_2 _was obtained from Police SA (Zaklady Chemiczne Police SA, Kuźnicka 1, Police, Poland). Acetylene 2.6 and argon 4.8 were purchased from Messer (Messer Polska sp. z o.o., Maciejkowicka 30, Chorzów, Poland). Phenol (99.5%, Sigma-Aldrich, Saint Louis, MO, USA) was used as a model organic pollutant. High purity water for the photocatalytic experiments and sample analysis was produced by Millipore Elix advantage water purification system (Millipore Corporation, Billerica, MA, USA).

### CVD growth of graphene

The CVD processes were performed in a horizontal furnace with a quartz tube reactor. Pristine TiO_2 _powder was placed in an alumina crucible. Prior to the synthesis, the reactor was evacuated down to 1 hPa, after which a temperature was increased to 400°C or 500°C in argon atmosphere (600 sscm). Then, acetylene was introduced also with 600 sscm. Six experiments at 400°C and 500°C for 10, 20, and 60 s have been performed, respectively. After each reaction, the furnace was cooled down to room temperature in argon atmosphere.

### Photocatalysts characterization

The morphology of the obtained material was characterized via transmission electron microscopy (FEI Tecnai F30, Frequency Electronics Inc., Mitchel Field, NY, USA). Raman spectra were acquired on the inVia Raman Microscope (Renishaw PLC, New Mills Wotton-under-Edge, Gloucestershire, UK) at an excitation wavelength of 785 nm. The surface properties of the photocatalysts were examined by means of Fourier transform infrared spectroscopy/diffuse reflectance spectroscopy (FTIR/DRS). Measurements were performed using a Jasco FTIR 4200 (Jasco International Co. Ltd., Hachioji, Tokyo, Japan) spectrometer equipped with a diffuse reflectance accessory (Harrick, Bridgewater, NJ, USA). Thermogravimetric analysis (TGA) was performed on the SDT-Q600 TGA (TA Instruments Inc., Milford, MA, USA) under an air flow of 100 mL/min and at a heating rate of 5°C/min in order to estimate a quantitative composition of the photocatalysts. The photocatalysts were characterized by UV-vis/DR technique using a Jasco V-650 spectrophotometer (Jasco International Co. Ltd., Hachioji, Tokyo, Japan) equipped with an integrating sphere accessory for diffuse reflectance spectra acquisition.

### Photoactivity evaluation

Photocatalytic activities of the samples under UV-vis (> 290 nm) irradiation were evaluated by oxidative decomposition of phenol under air in aqueous solutions. The reaction was performed as described in previous publication [16], while the photocatalytic activity under visible light was tested during 24 h phenol photooxidation processes. A photocatalyst (50 mg) was suspended in an aqueous solution (250 cm^3^) containing 10 ppm phenol solution, the magnetic stirring was fixed at 500 rpm. Photodecomposition process was performed using halogen lamp with a power of 70 W (Philips Electronics North America Corporation, New York, NY, USA). The light photoirradiation (> 420 nm) was performed using a cutoff filter (Hoya Y44 Tokina Co. Ltd., Saitama, Japan) to eliminate UV light. The radiation intensity was of about 883 W/m^2 ^vis. The concentration of phenol in the solution as well as the concentration of total organic carbon (TOC) remained in the solution after the photodegradation process was measured. Prior to all the measurements of the phenol concentration, the solution was filtered through a membrane filter with 0.45 mm pore diameter.

## Results and discussion

### Characterization of the photocatalysts

**Figure 1:**
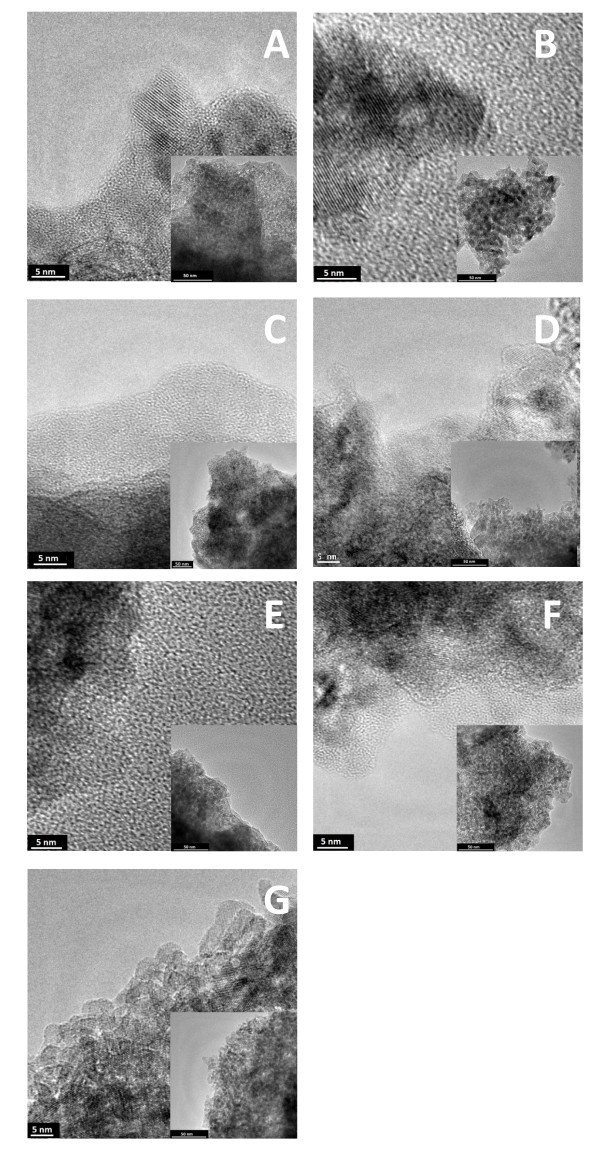
**TEM images of T-G-400-10 (A), T-G-400-20 (B), T-G-400-60 (C), T-G-500-10 (D), T-G-500-20 (E), T-G-500-60 (F) and starting TiO_2 _(G)**.

Figure 1 presents transmission electron microscopy (TEM) images of the CVD-treated TiO_2_. One can notice that the surface of TiO_2 _nanocrystals is covered with a carbon layer. On the basis of the detailed analysis, it was found that the thickness of the external layer increases with the extension of the CVD treatment time. The shape of the nanocrystals remained unchanged in respect to the starting material (see insets of Figure 1).

**Figure 2:**
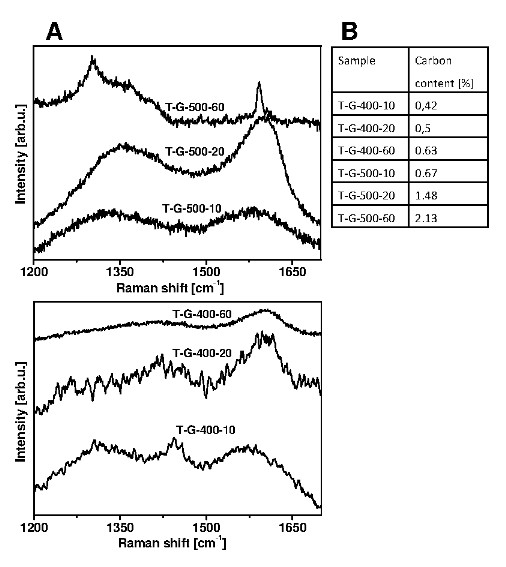
**Raman spectra (A) and carbon content in the catalysts (B)**.

For further characterization, Raman spectroscopy was utilized. Figure 2A presents Raman spectra of TiO_2 _after CVD processes. The spectra exhibit two peaks: the G band at approximately 1,582 cm^-1 ^arising from the in-plane vibration of sp^2 ^carbon atoms and is a doubly degenerated phonon mode (*E*_2g _symmetry) at the Brillouin zone center, and the D band at around 1,350 cm^-1 ^is a breathing mode of *A*_1*g *_symmetry involving phonons near the *K *zone boundary and arises from structural defects such as presence of pentagons and heptagons in carbon lattice [17]. These observations confirm the graphitic character of the grown carbon on the TiO_2 _surface. The spectra of the samples being in CVD reactor at 400°C reveal additional peak at 1,440 cm^-1 ^correlated to amorphous carbon. This is in agreement with the TEM analysis. In order to estimate the carbon content in the samples, TGA was employed. The results are presented in the table in Figure 2B. The increase of the CVD process time is strongly correlated with the carbon deposit weight.

**Figure 3:**
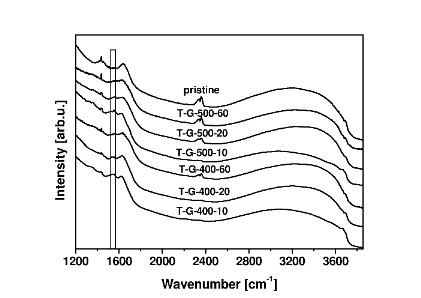
**FT-IR spectra of pristine TiO_2 _and modified photocatalysts**.

The surface properties of the photocatalyst were investigated by FTIR/DRS (Figure 3). It can be seen that the new band at 1,546 cm^-1 ^is present in all modified catalysts. These band can be attributed to aromatic C = C stretch [18], which suggests that carbon incorporation into the structural lattice was successful. One can notice that this band becomes broader with increasing reaction time for both temperatures. Furthermore, it is worth noticing that no band appears at approximately 3,000 cm^-1^, assigned to C-H group, suggesting the complete decomposition of acetylene during CVD. These results fully confirm analyses of TEM and Raman spectroscopy.

Finally, UV-vis/DR spectroscopy (not presented here) was used in order to estimate band-gap energy (*E*g) of the samples calculated using the Kubelka-Munk model [19]. In Table 1 the band-gap energy values of the samples are given. It was found that stronger photon absorption, thus the lower *E*g values, was obtained in the specimens prepared with longer time of deposition at higher temperature. Nevertheless, it is worth noting that the visible light absorption itself is not tantamount to enhanced photocatalytic activity since the photoproduced electrons and holes in TiO_2 _may experience a rapid recombination which diminishes the visible light utilization and as a result of the efficiency of the photocatalytic reaction significantly.

**Table 1:** *E*g values and photocatalytic activity under UV-vis and visible light irradiation

	*E*g	UV-vis light irradiation		Visible light irradiation
Photocatalyst	(eV)	Rate constant - *k*	TOC removal (%)	TOC removal (%)
Pristine	3.30	0.0061	59	4.0
T-G-400-10	3.18	0.0021	14	8.7
T-G-400-20	3.22	0.0019	14	4.6
T- G-400-60	2.99	0.0016	11	2.1
T-G-500-10	3.14	0.002	21	9.3
T-G-500-20	3.06	0.0016	15	5.4
T-G-500-60	2.9	0.0011	10	5.2

### Photocatalytic activity of the materials

The photocatalytic activity of new materials was tested as described in experimental section and results are presented in Table 1. The phenol degradation kinetics under UV-vis and visible light were pseudo first-order, the rate constants (*k*) were obtained by fitting the experimental data. Functionalization of TiO_2 _with graphitic carbon resulted in the increase of photoactivity under visible light, whereas the UV-vis light activity decreased in comparison to starting material. Under visible light irradiation, TOC removal rate increased from 4% for pristine material to ca. 9% for samples heated at 400°C or 500°C with very short (10 s) deposition time. This relation is almost identical for both types of irradiations. Thus, the photoactivity decreases in samples being treated longer in CVD. Additionally, the effect of photoactivity is also more pronounced in case of materials prepared at 500°C in comparison to the materials treated at 400°C. These results are in agreement with their values of *E*g listed in Table 1. As it was mentioned above, the lower band-gap energy is not a key factor for the visible light applications. The photocatalytic activity depends on the efficiency of utilization of the fraction of the incident radiation absorbed by the catalyst, which results in the generation of electron/hole couples. These couples, though, may undergo a rapid recombination, resulting in neglecting quantum efficiency. Nevertheless, in case of presented materials, it is observed that higher visible light absorption and lower *E*g values indicate higher visible light photoactivity. Thus, these results prove, which was earlier predicted by other researchers [20], that recombination of electron/hole can be retarded by graphene. Lee et al. [11] described in details the reasons of graphene recombination inhibition. Graphene may work as electron acceptor and transporter. Due to its two-dimensional π-conjugation structure, it may also work as an acceptor of photogenerated electrons. On the other hand, due to its high conductivity, effective charge separation may be accomplished through electrons transport. It is also proved here that the carbon layer can also consist of thin layer of graphite and the above described effects are still observed.

## Conclusions

In summary, we have successfully modified TiO_2 _nanocrystals with graphitic carbon via CVD. Here, acetylene was used as a carbon feedstock and TiO_2 _as a substrate. The investigations indicate that higher CVD temperature and longer time of reaction resulted in enhanced deposition of carbon. The amount of deposited carbon layer blue shifted the band-gap energy of the samples in comparison to the pristine TiO_2_. Photocatalytic activity of the materials was explored and it was found that TiO_2 _coated by thin graphitic layer exhibits higher photoactivity under visible light and lower activity under UV-vis light irradiation. Interestingly, samples being treated in CVD for a shorter time and higher temperature showed significantly better activity in the visible region.

## Competing interests

The authors declare that they have no competing interests.

## Authors' contributions

MW carried out CVD processes, Raman spectroscopy analysis and TGA analysis. DD carried out photocatalytic processes of phenol decomposition, FTIR/DRS analysis, UV-vis/DR analysis and TOC analysis. AM supervised analyses of the materials and preparation of the manuscript. EM performed TEM analysis and supervised analyses of the materials and preparation of the manuscript. All authors read and approved the final manuscript.
